# Construction Notes

**Published:** 1894-02-10

**Authors:** 


### CONSTRUCTION NOTES.

**New Wing of the Staffordshire General Infirmary.**

This new north wing has recently been opened. The arrangements of the building are simple and complete, and the contractor has apparently done his work solidly and well. It has a total frontage of about eighty-five feet to the main street, and is a lofty two-storied building, being erected on an under structure of arches, themselves springing from a cemented foundation, air thus circulating freely underneath with no possibility of damp striking upwards. The front elevation is of plain design, being of red brick and cement stucco. The building is connected to the old infirmary by a covered passage, and can be entered also from the grounds at that end by a staircase. There are two wards, one above and one below, each containing fourteen beds, so placed that there is a window between each bed. The arrangements for ventilation are excellent. At the end of the wards, remote from the old building, are the closets, bath-rooms, and lavatories, and noticeable here is McHardy's patent apparatus for the cleansing of hospital appliances. At the end of the old building adjoining the infirmary are ward kitchens thoroughly equipped. The architect was Mr. Aston Webb; the tender of Mr. F. Espley, of Stafford, for the erection of the new wing being accepted at £3,200, together with that of Mr. Clement Jeakes, engineer, for the heating apparatus and other mechanical work.

### New Cottage Hospital at Fleetwood.

This new hospital will be an ornament to the town, as well as a most useful institution. The building itself will be plain and good-looking. Messrs. Drummond and Sons, of Fleetwood, prepared the plans, and their tender (£2,200) was also accepted for the work. The elevations will be carried out in red brick and red stone dressings. The main entrance will face due south. There will be a committee-room on the right 17 ft. by 13 ft., and on the left a matron's room of a similar size. On the same floor there will be two rooms 13 ft. by 12 ft., which will be used as a bed-room and a bath-room. Two wings will project from the main building, and these will be converted into wards, the size being 28 ft. by 9 ft., each containing four beds, with lavatory, w.c., &c. The surgery-room is 12 ft. by 9 ft., the operating-room efficient and well-appointed, and in the rear there is an ample kitchen, scullery, and wash-house accommodation. The entrance at the back will be close to the operating and surgery rooms, in order that patients may reach their rooms without going through the front entrance. Upstairs there are three good bed-rooms, and two wards for two patients each. The whole of the sanitary arrangements will be carried out upon the latest system, particular attention being paid to this point.

### Glasgow Victoria Infirmary, New Pavilion.

This new pavilion, which has been erected in extension of the Victoria Infirmary, was recently opened by the Lord Provost. By this addition the accommodation of the infirmary will be more than doubled, so that, under normal conditions, there may now be housed and medically or surgically treated as many as 150 patients. The principal portion of the new extensions of the infirmary is a pavilion, in which, in addition to the basement floor, there are three wards which occupy the floors above, and each will be fitted up for 18 adult patients. The new pavilion, which runs almost due north and south, is connected with the old buildings by means of a long corridor. The new wards are constructed on the same general plan as those forming the front pavilion, which extends southwards from the main block of buildings which forms the administrative department, and in which is contained the board-room. Besides the wards proper, which are very spacious apartments, there are a number of smaller side rooms adjoining the wards, each of which has accommodation for three or four beds. These side rooms are somewhat larger than the corresponding rooms in the pavilion which was first erected. In the new pavilion there is also a dining-room for the nurses, together with a room for the resident doctors; and in the basement of this pavilion there is an isolation-room, in which patients will be received whose condition is of a suspicious nature, for no cases of infectious disease are knowingly received into the infirmary. A new operating theatre is also included in the extension. Messrs. Campbell, Douglas, and Morrison, Glasgow, are the architects, and Mr. Thomas Young is the consulting engineer for the heating, lighting, and ventilating arrangements.

